# NLRC5, a promising new entry in tumor immunology

**DOI:** 10.1186/s40425-016-0143-z

**Published:** 2016-07-19

**Authors:** Sonia T. Chelbi, Greta Guarda

**Affiliations:** Department of Biochemistry, University of Lausanne, Epalinges, 1066 Switzerland

**Keywords:** NLRC5, MHC class I, Tumor immunogenicity, Immunotherapy, Interferons

## Abstract

The recent use of T cell-based cancer immunotherapies, such as adoptive T-cell transfer and checkpoint blockade, yields increasing clinical benefit to patients with different cancer types. However, decrease of MHC class I expression is a common mechanism transformed cells take advantage of to evade CD8^+^ T cell-mediated antitumor responses, negatively impacting on the outcome of immunotherapies. Hence, there is an urgent need to develop novel approaches to overcome this limitation.

NLRC5 has been recently described as a key transcriptional regulator controlling expression of MHC class I molecules. In this commentary, we summarize and put into perspective a study by Rodriguez and colleagues recently published in Oncoimmunology, addressing the role of NLRC5 in melanoma. The authors demonstrate that NLRC5 overexpression in B16 melanoma allows to recover MHC class I expression, rising tumor immunogenicity and counteracting immune evasion. Possible ways of manipulating NLRC5 activity in tumors will be discussed. Highlighting the therapeutic potential of modulating NLRC5 levels, this publication also encourages evaluation of NLRC5, and by extension MHC class I pathway, as clinical biomarker to select personalized immunotherapeutic strategies.

## Background

Major histocompatibility complex (MHC) class I display is a fundamental defense mechanism enabling CD8^+^ T lymphocytes to identify and kill infected or transformed cells. Loss or down-regulation of MHC class I expression, accompanied by impaired expression of one or several components of the antigen processing machinery (APM), is frequently observed in many solid and hematopoietic tumors [1]. Therefore, these processes constitute common mechanisms malignant cells take advantage of to evade natural or immunotherapy-induced immune responses [2]. At the molecular level defective expression of MHC class I and APM components result from either “soft/reversible” alterations driven by epigenetic and posttranscriptional mechanisms or “hard/irreversible” structural genetic alterations, such as mutations or deletions [1, 2].

It recently became clear that the nucleotide-binding oligomerization domain (NOD)-like receptor (NLR) family member NLRC5 (NLR caspase recruitment domain containing protein 5) is a key transcriptional regulator of the MHC class I pathway [3–7]. *Nlrc5* knockout mouse models demonstrated the crucial role of this NLR to maintain high MHC class I expression in several cell types and most prominently in lymphocytes [4–7]. Both in human and mouse, NLRC5 has a very focused transcriptional regulatory activity, controlling expression of classical and selected non-classical MHC class I genes, and few genes coding for APM proteins [3, 5]. Mechanistically, this specificity is achieved through the formation of a unique transcriptional regulatory complex on the promoter of the target genes [3, 5, 8, 9].

We previously reported that *Nlrc5*^-/-^ lymphoid targets are killed less efficiently than controls by cytotoxic T cells, due to their low expression of MHC class I [4]. Moreover, low NLRC5 levels were observed in certain lymphoid tumor-derived cell lines [4]. These results suggested that downmodulation of NLRC5 constitutes a mechanism of tumor immunoevasion.

### Role of NLRC5 in B16 melanoma

The dedicated activity of NLRC5 to transactivate MHC class I and APM genes renders this NLR particularly interesting to study in the context of tumor development, immunity, and escape. A recent study by Rodriguez et al. published in Oncoimmunology presents the first experimental proof for the importance of NLRC5 in counteracting tumor immune evasion [10]. The authors generated a B16 melanoma cell line stably expressing NLRC5. B16 cells exhibit high mutation rate, as melanoma in general, and reversible downregulation of the MHC class I-mediated antigen presentation pathway, being therefore well suited to test the immunogenic effects of NLRC5 [11–14]. As expected, NLRC5-expressing cells showed strongly increased MHC class I display and augmented transcript levels of selected APM factors. These cells were effective in presenting exogenous and – to some extent – endogenous tumor antigens (TAs), as shown by the activation of Pmel-1 TCR transgenic CD8^+^ T cells, which are specific for the melanoma immunogenic epitope gp100_25-33_. Along this line, NLRC5-expressing B16 cells formed smaller tumors following subcutaneous implantation and fewer foci in the lung upon intravenous injection into C57BL/6 hosts. Whereas tumor growth was significantly hindered when grafted into wild type mice, this was not observed in immunodeficient or CD8^+^ T cell-depleted hosts, indicating that NLRC5-expressing tumor cells evoked a protective antitumoral cytotoxic T cell response. A possible drawback of this set-up, is the choice of expressing human NLRC5 in B16 cells, as it only shares partial identity to the murine homolog and could therefore introduce exogenous epitopes. However, the superior activation of Pmel-1 T cells by NLRC5-expressing B16 melanoma strongly supports the idea that the enhanced capacity to present tumor antigens is the genuine cause for the immunogenicity of this cell line.

Interestingly, the authors also tested the use of B16 melanoma cells stably expressing NLRC5 in combination with the co-stimulatory molecule CD80 [10]. Whereas in vitro the additional presence of CD80 led to a better immune response, this setting was less efficient in controlling tumor growth and spreading in vivo. Possibly, the cause for this counterintuitive result lies in the interaction of CD80 with the inhibitory receptor cytotoxic T-lymphocyte-associated protein 4 (CTLA-4) expressed on tumor infiltrating T cells, suggesting that co-treatment with CTLA-4 blocking agents could specifically boost antitumor immunity in this context.

Increasing NLRC5 expression in tumor cells can thus enhance natural anticancer immunity and immunotherapeutic responses especially in the case of MHC class I low tumors. As proof of principle, the authors immunized mice with irradiated B16 melanoma cells expressing NLRC5. In a prophylactic setting, this treatment effectively protected mice against parental B16 cell engraftment. Taken together, these data underline the importance of NLRC5 in rising tumor immunogenicity and limiting tumor immune escape.

### Perspectives

**Fig. 1:**
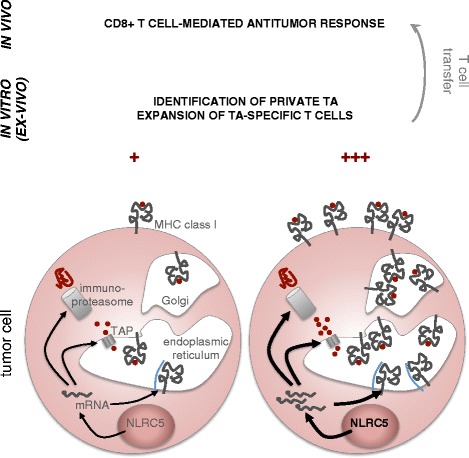
Potential applications of NLRC5 in immunotherapy. NLRC5 is a specific transactivator of MHC class I and a subset of APM genes. An increase or recovery of NLRC5 expression in tumor cells could therefore improve tumor antigen (TA) presentation and boost the CD8^+^ T cell-mediated antitumor response. Manipulation of NLRC5 expression could be envisaged in vitro/ex vivo and/or directly in patients. In vitro, and anyway in the case of irreversible mutations, gene transfer strategies might be considered. Alternatively, type I IFN is known to raise NLRC5 expression. These approaches will facilitate identification of private TAs by mass spectrometric analysis and expansion of TA-specific T cell clones in the perspective of transfer therapies. In addition, type I IFN could be administrated to patients, possibly in combination with chemotherapy, radiations, or checkpoint inhibitors, in order to increase tumor immunogenicity and improve the overall therapeutic benefit

One way of translating these results to human therapy envisages engineering patient-derived tumor cells to overexpress NLRC5 in vitro. This would facilitate identification of private tumor epitopes and expansion of endogenous antitumor T cells (Fig. 1). Obviously, such an approach will be feasible only if the MHC class I pathway of the tumor is devoid of structural alterations, in which case more complex gene transfer strategies should be considered.

Data by Rodriguez and colleagues demonstrate the beneficial impact of increasing the activity of NLRC5 in tumor cells in vivo (Fig. 1) [10]. The effects of NLRC5 correlate with its expression levels, which can be enhanced by type I and II interferon (IFN), as shown also for melanoma cells [3, 4, 6, 7, 9, 10, 15]. Provided that *NLRC5* and its target genes are devoid of irreversible genetic defects, treatment with type I IFN should therefore represent a way of raising the levels of NLRC5 and the immunogenicity of cancer cells. IFN has already shown potential in tumor treatment [2, 16–18], and would have the additional benefit of inducing NLRC5 in professional antigen-presenting cells and of lowering the threshold for natural killer (NK) cell activation [19, 20]. It might also be of interest to study NLRC5 levels and activity in conditions relevant to the clinic, such as chemotherapy or radiation, which have been shown to induce an IFN response [16, 17].

Only recently, with the advent of the so-called “checkpoint inhibitor” and adoptive T cell transfer therapies, we fully appreciate the power of the antitumoral cytotoxic immune response [21, 22]. However, immune evasion mechanisms targeting antigen presentation by transformed cells remain a major obstacle to such approaches. The role of endogenous NLRC5 with regard to tumor progression and immunotherapy efficacy is still unexplored; *Nlrc5*-knockout mouse models are valuable tools to address these points, in addition to a thorough analysis of *NLRC5* transcript levels and somatic mutations in human tumors. Indeed, data reporting *NLRC5* alterations are starting to emerge [23–26], showing that mutations and copy number variations are common across various tumor types. In how far these are passenger or driver mutations needs to be determined and will be crucial for our understanding of tumor immunobiology and for meeting clinical decisions on the use of immunotherapies. We therefore need to gain a comprehensive view of the mechanisms controlling antigen presentation and the ways to influence them. As anticipated in the study by Rodriguez and colleagues, NLRC5, given its central role in regulating the MHCI pathway, shows promise to improve immunotherapies.

## Abbreviations

APM, antigen processing machinery; CTLA-4, cytotoxic T-lymphocyte-associated protein 4; IFN, interferon; MHC, major histocompatibility complex; NK, natural killer; NLRC5, NLR caspase recruitment domain containing protein 5; NLR, NOD-like receptor; NOD, nucleotide-binding oligomerization domain; TA, tumor antigen; TCR, T cell receptor
